# Expression of GLUT4 and FAP in urothelial bladder carcinoma: correlation with angiogenesis and clinicopathological characteristics

**DOI:** 10.1186/s43046-022-00145-0

**Published:** 2022-09-26

**Authors:** Marwa A. Abd El-Azeem, Mona A. Ali, Safinaz H. El-Shorbagy

**Affiliations:** Pathology Department, Faculty of Medicine, Tanta University, Tanta, Egypt

**Keywords:** Angiogenesis, Clinicopathological characteristics, FAP, GLUT4, Immunohistochemistry, Urothelial carcinoma

## Abstract

**Background:**

Urothelial carcinoma (UC) is the most common type of bladder cancer. Glucose transporter 4 (GLUT4) is one of glucose transporter proteins’ family which facilitates glucose transport inside the cells. It was found to be overexpressed in several malignant tumors. Cancer-associated fibroblasts (CAFs) are heterogeneous stromal cells located adjacent to cancer cells and are considered one of the most important tumor stromal cells. They have been associated with enhancing tumor growth and invasion. GLUT4 expression in malignant epithelial cells and fibroblast activation protein (FAP) expression in CAFs of UC in relation to angiogenesis and clinicopathological characteristics are studied in this work.

**Materials and methods:**

The study was carried out on 72 paraffin blocks of UC (27 radical cystectomies and 45 transurethral resections). Immunohistochemical staining was performed with GLUT4, FAP, and CD34 antibodies. Expression of GLUT4 and FAP was classified according to the staining intensities and percentages into low and high groups. CD34-stained microvessels’ mean count in five microscopic fields (×200) was taken as the microvessel density (MVD).

**Results:**

GLUT4 overexpression was detected in 32 UC. It was significantly associated with high-grade tumors, advanced primary tumor (pT) stage, lymphovascular invasion (LVI), and regional lymph node invasion. High FAP expression was appreciated in 27 UC and was significantly linked to LVI and advanced TNM staging. Intratumor MVD significantly increased in UC with muscle invasion, LVI, and regional lymph node and/or distant metastasis. A significant positive correlation between GLUT4, FAP expression, and MVD was found.

**Conclusion:**

GLUT4 and FAP expression was significantly associated with increased intratumor MVD and adverse clinicopathological factors.

## Background

Bladder cancer (BC) is the most common malignancy involving the genitourinary tract and is one of the leading causes of cancer-related death worldwide. It affects males more than females [1]. In Egypt, BC is the third most common malignant tumor. The incidence of newly diagnosed cases in 2020 in both sexes was 7.9%. Males’ and females’ incidences were 22.5% and 5.2%, respectively [2].

Urothelial carcinoma (UC) is the most common histologic type, constituting more than 90% of BC cases [3].

Despite improvement in the treatment of UC, many patients still suffer from disease recurrence or metastasis [4]. Therefore, understanding the mechanisms underlying tumor invasion, metastasis, and treatment resistance are important to improve the clinical outcome of UC patients.

Uncontrolled proliferation is an important feature of cancer. To maintain this proliferation, cancer cells must increase demands for energy production. Otto Warburg observed that tumor cells convert glucose into lactate by aerobic glycolysis, even in the presence of oxygen, instead of the oxidative phosphorylation process for energy production. This is known as the “Warburg effect.” It is termed aerobic glycolysis to differentiate it from anaerobic glycolysis, which occurs in the absence of oxygen, indicating that the cancer cell metabolism of glucose is not influenced by the lack of oxygen [5].

Owing to the hydrophilic nature of glucose, it needs specific carrier proteins to cross the plasma membrane of cells. Two families of membrane-associated carriers transport glucose into the cell: the facilitative glucose transporter (GLUT) proteins and the sodium-coupled glucose co-transporter (SGLT) proteins. GLUTs move sugars down a concentration gradient, while SGLT proteins require energy for glucose transport [6].

GLUT4 (SLC2A4), a class I member of GLUTs, is a high-affinity glucose transporter that is normally expressed in insulin-dependent tissues like the adipose tissue, skeletal muscle, and myocardium [7]. GLUT4 is present in intracellular vesicles (GLUT4-storage vesicles) which can be transported to the plasma membrane in response to its activation. Several studies showed that GLUT4 was expressed and amplified in breast [8], prostatic [9], and other carcinomas [10], and this expression was associated with invasiveness and poor prognosis of these cancers suggesting that it might play a role in process of tumorigenesis.

The malignant tumor tissue is composed of two elements: cancer cells and non-cancerous stromal elements called tumor-associated stromal cells. These tumor-associated stromal cells include a variety of cells such as macrophages, endothelial cells, and fibroblasts. They play an important role in the tumor microenvironment facilitating the growth, migration, and metastasis of cancer cells [11].

Most solid epithelial tumors have desmoplastic stroma consisting mainly of activated fibroblasts also named cancer-associated fibroblasts (CAFs). Tumors with excessive desmoplasia are called stroma high tumors which were found to have a poor prognosis [12]. CAFs are heterogeneous cells regarding their biological function in enhancing cancer development and progression [13]. They are located adjacent to cancer cells and are considered one of the most important tumor stromal cells. Their existence in the stromal microenvironment was found to enhance tumor growth, induce angiogenesis, and promote metastasis via remodeling of the extracellular matrix (ECM) [14].

Different fibroblast labeling markers including alpha-smooth muscle actin (ASMA), platelet-derived growth factor receptor-alpha and -beta (PDGFR α, -ß), fibroblast specific protein (FSP), and fibroblast activation protein (FAP) have been studied to identify CAFs and to explore their roles in malignant tumors’ proliferation and invasion as prognostic markers [15].

Fibroblast activation protein (FAP-α, also called seprase) is a prolyl-specific serine proteinase that is expressed in fibroblasts. High FAP expression has been considered a biomarker for tumor progression and metastasis in numerous types of malignant tumors [16].

As far as we know, the expression of GLUT4 in UC and its relation to FAP expression and angiogenesis have not been previously studied, so this work aims to study the GLUT4 expression in malignant epithelial cells and FAP expression in CAFs of UC in relation to angiogenesis and clinicopathological characteristics.

## Methods

After ethical approval by the local ethical committee, Faculty of Medicine (34370/1/21), 72 primary UC paraffin tissue blocks were collected retrospectively from the archive of the Pathology Department. Received specimens were either of TURBT (transurethral resection of bladder tumor) or radical cystectomy with lymphadenectomy. Cases with recurrent tumor, preoperative radiation or chemotherapy, extensive tumor necrosis, or missing clinical data were excluded from the study.

Clinical data of the cases was obtained from the datasheets of the patients kept in the records of the Pathology Department which include the patient’s age, gender, primary or recurrent disease, type of surgical procedure performed, and the assessment of distant metastasis prior to radical cystectomy.

Afterward, H&E-stained sections from the 72 relevant paraffin blocks (27 radical cystectomies and 45 TURBT) were prepared and examined to determine tumor grade, primary tumor stage (pT), associated carcinoma in situ component (T_is_), presence of lymphovascular invasion (LVI), and regional lymph node invasion (only in specimens of radical cystectomy with lymphadenectomy).

Stages of pT_a_, pT_1_, and pT_2a_ were evaluated in TURBT and the stage of pT_2b_ was evaluated in radical cystectomy specimens. pT_a_ (non-invasive) and pT_1_ (UC with lamina propria invasion) are considered superficial tumors, whereas pT_2a_ (invasion of the inner half of muscularis propria) and pT_2b_ (outer half muscle invasion) are considered muscle-invasive tumors. UC was graded into low- and high-grade tumors as defined by the 2004 WHO grading system [17] and staged according to the TNM staging system [18].

### Immunohistochemistry

Five-μm-thick sections were prepared from paraffin-embedded tissue blocks. After deparaffinization with xylene, the sections were rehydrated using decreasing grades of ethanol solutions. For antigen retrieval, the tissue sections were incubated in 0.01 M citrate buffer (pH 6.0). Endogenous peroxidase was blocked via incubation in 3% hydrogen peroxide for 5 min. Overnight incubation at 4°C with GLUT4 polyclonal antibody (1:100; ABclonal, USA), FAP polyclonal antibody (1:50; ABclonal, USA), and CD-34 polyclonal antibody (1:150; ABclonal, USA) was performed. The slides were then counter-stained with Mayer’s hematoxylin. Negative control was performed by processing sections without the primary antibodies.

### Evaluation of immunohistochemical staining

Evaluation of immunohistochemical staining was carried out by three pathologists blinded to the clinicopathological characteristics and clinical outcomes of the patients.

#### GLUT4 IHC evaluation

Positive GLUT4 protein expression was identified as membranous or cytoplasmic brown staining. The quantitative estimation of GLUT4 expression was performed by counting the number of positive cells in 5 randomly selected fields (400×) as follows: 0% of cells positive (-, negative), < 25% of positive cells (+, weakly positive), 25–50% of positive cells (++, moderately positive), and > 50% of positive cells (+++, strongly positive). Negative and weakly positive were considered as low expression, while moderately and strongly positive were evaluated as a high expression [19]. The skeletal muscles were used as a positive control.

#### FAP IHC evaluation

FAP expression was examined in the stromal fibroblasts adjacent to neoplastic epithelial nests (CAFs). FAP immunoreactive cells were counted in 5 microscopic fields (400×). The percentage scoring of positive cells was as follows: 0 (0–5%), 1 (6–25%), 2 (26–50%), 3 (51–75%), and 4 (>75%). The staining intensity was scored and categorized as follows: (0 =negative, 1 =weak, 2 =moderate, and 3 =strong). A final score was obtained by multiplying the percentage and the intensity scores. 0: negative (-), 1–4: weakly positive (+), 5–8: moderately positive (++), and 9–12: strongly positive (+++). Moderate and strong positivity were considered a high expression group, while negative and weak positivity were considered a low expression group [20].

#### Determination of microblood vessel density (MVD)

Microvessels were defined as any CD34-positive brown cytoplasmic staining of endothelial or endothelial cell clusters with or without a visible lumen. The stained sections were first viewed at low magnification (×100) to identify the areas with the highest number of microvessels in the tumoral zone (hot spots). Stained microvessels were counted in five microscopic fields (×200), and the mean number was considered as the microvessel density (MVD). Large vessels with thick muscular walls were excluded from counting [21].

### Statistical analysis

Statistical analysis was performed using SPSS (version 20, IBM, Armonk, NY, USA). The mean and standard deviation were calculated for MVD and the patient’s age. The comparison of MVD in relation to clinicopathological characteristics was done using the *t*-test for two unpaired samples. Chi-square (*χ*^2^) test was used to determine the significance of the association between FAP and GLUT4 expression and the clinicopathologic characteristics of the studied cases. The correlation between biomarkers’ expression was done by Spearman’s correlation (*r*). The results were considered statistically significant if the *p* value was < 0.05.

## Results

Table 1 summarizes the clinicopathological characteristics of the studied cases. The age of patients ranged from 45 to 74 years with a mean age of 64.04±7.45, the majority of patients were ≥ 60 years (70.8%). There was a male predominance (61.1%). Twenty-five tumors (34.7%) were low-grade and 47 were high ones (65.3%). A pT_a_ UC was found in 20 cases (27.8%), pT_1_ was detected in 25 cases (34.7%), and T_2a_ and T_2b_ were appreciated in 13 (18.1%) and 14 (19.4%) tumors, respectively. Associated carcinoma in situ (T_is_) was recognized in 29 tumors (40.3%) and LVI in 33 tumors (45.8%). Out of 27 radical cystectomies with regional lymphadenectomy specimens, lymph node invasion was found in 15 cases (55.6%) and distant metastasis was reported in 4 cases (14.8%).

**Table 1:** Clinicopathological characteristics of studied cases

Variables	Number (%)
**Age**	
**Mean±SD**	64.04**±**7.45
<60	21 (29.2)
≥60	51 (70.8)
**Gender**	
Male	44 (61.1)
Female	28 (38.9)
**Tumor grade**	
Low	25 (34.7)
High	47 (65.3)
**T-Primary tumor**	
pT_a_	20 (27.8)
pT_1_	25 (34.7)
pT_2a_	13 (18.1)
pT_2b_	14 (19.4)
**Associated T**_**is**_	
Absent	43 (59.7)
Present	29 (40.3)
**LVI**	
Absent	39 (54.2)
Present	33 (45.8)
**N-Regional lymph nodes**	
N_0_	12 (44.4)
N_1_	10 (37.1)
N_2_	5 (18.5)
**M - Distant metastasis**	
M_0_	23 (85.2)
M_1_	4 (14.8)

### The relation between GLUT4 expression and clinicopathological characteristics

**Fig. 1:**
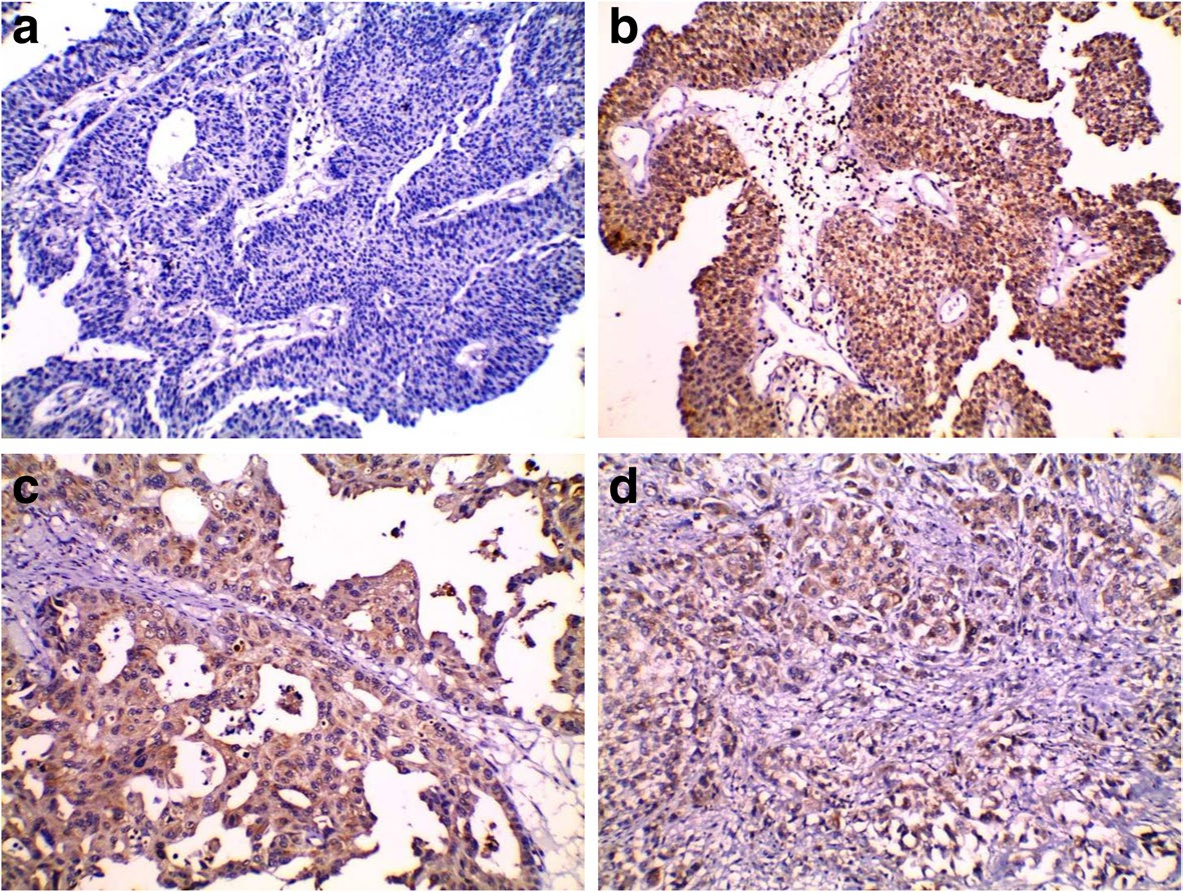
Immunohistochemical expression of GLUT 4 (×200) shows: **a** low GLUT4 expression in low-grade pT_a_ urothelial carcinoma (UC), **b** high GLUT4 expression in low-grade pT_a_ UC, **c** high GLUT4 expression in high-grade pT_1_ UC, and **d** high GLUT4 expression in high-grade pT_2_ UC

High cytoplasmic GLUT4 immunohistochemical expression was identified in 40/72 UC specimens (44.4%). This high expression was significantly associated with high-tumor grade as 55.3% of high-grade tumors exhibited GLUT4 overexpression compared to 24% of low-grade ones (*p*=0.013). Also, the expression of GLUT4 significantly increased with increased depth of tumor invasion, in which pT_2b_, pT_2a_, and pT_1_ carcinomas (85.7%, 46.2%, and 40%, respectively) showed a higher rate of GLUT4 overexpression than pT_a_ (20%) (*p*=0.002). In addition, high GLUT4 expression was significantly more detected in tumors with LVI (63.7%) than those without (28.2%) (*p*=0.004). Similarly, UC with regional lymph node invasion significantly showed a higher GLUT4 expression than tumors without lymph node invasion (N_1_; 60% and N_2_; 80%, *p*=0.026). However, no statistically significant difference regarding high GLUT4 expression with the age of patients (*p*=0.797), gender (*p*=0.234), presence of associated T_is_ (*p*=0.227), or M stage (*p*=0.065) was detected (Table 2, Fig. 1).

**Table 2:** The relation between GLUT4 expression and clinicopathological characteristics

Variables	GLUT 4 expression		***x***^***2***^	***p*** value
	Low ***n***=40 (55.6)	High ***n*** = 32 (44.4)		
**Age**				
<60	11 (52.4)	10 (47.6)	0.121	0.797
≥60	29 (56.9)	22 (43.1)		
**Gender**				
Male	27 (61.4)	17 (38.6)	1.546	0.234
Female	13 (46.4)	15 (53.6)		
**Tumor grade**				
Low	19 (76)	6 (24)	6.483	0.013^*^
High	21 (44.7)	26 (55.3)		
**T-Primary tumor**				
pT_a_	16 (80)	4 (20)		
pT_1_	15 (60)	10 (40)	14.713	0.002^*^
pT_2a_	7 (53.8)	6 (46.2)		
pT_2b_	2 (14.3)	12 (85.7)		
**Associated T**_**is**_				
Absent	21 (48.8)	22 (51.2)	1.952	0.227
Present	19 (65.5)	10 (34.5)		
**LVI**				
Absent	28 (71.8)	11 (28.2)	9.088	0.004^*^
Present	12 (36.4)	21 (63.7)		
**N-Regional lymph nodes**				
N_0_	10 (76.9)	2 (23.1)		
N_1_	4 (40)	6 (60)	7.290	0.026^*^
N_2_	1 (20)	4 (80)		
**M - Distant metastasis**				
M_0_	18 (78.3)	5 (21.7)	4.636	0.065
M_1_	1 (25)	3 (75)		

*LVI* lymphovascular invasion, *x*^2^ chi-square test, *p <0.05

### The relation between FAP expression and clinicopathological characteristics

**Fig. 2:**
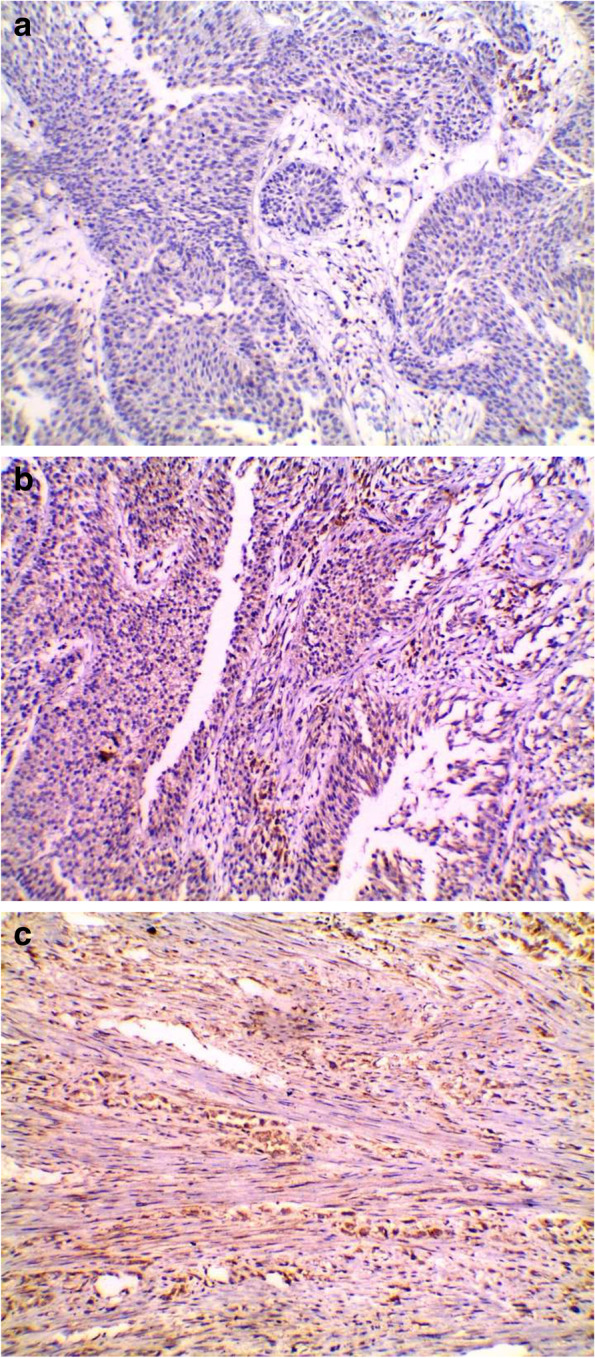
Immunohistochemical expression of FAP (×200) shows: **a** low FAP expression in cancer-associated fibroblasts (CAFs) in low-grade pT_a_ UC, **b** high FAP expression in CAFs in low-grade pT_1_ UC, and **c** high FAP expression in CAFs in high-grade pT_2_ UC

FAP immunohistochemical cytoplasmic expression (either low or high) was examined only in stromal fibroblasts adjoining the tumor cells (CAFs). FAP expression in epithelial neoplastic cells was overlooked. High FAP immunostaining was detected in 27 tumors (37.5%). Muscle invasive pT_2a_ (61.5%) and pT_2b_ UC (71.4%) significantly showed high FAP expression more often than non-muscle-invasive pT_a_ (15%) or pT_1_ UC (24%) (*p*=0.001). Also, tumors with LVI (51.5%) significantly exhibited high FAP immunolabeling in CAFs compared to those without (25.6%) (*p*=0.030). FAP expression was significantly associated with a higher N stage and the presence of distant metastasis (*p*=0.041 and 0.004, respectively). Although high FAP immunolabeling was noticed more with high-grade UC (42.6%) than low-grade ones (28%), however, this difference did not reach statistical significance (*p*=0.308). Also, high FAP expression in CAFs did not significantly relate to the patient’s age, gender, or presence of associated in situ component (*p*=0.790, 0.081 and 0.805, respectively) (Table 3, Fig. 2).

**Table 3:** The relation between FAP expression and clinicopathological characteristics

Variables	FAP expression		***x***^***2***^	***p*** value
	Low ***n***= 45 (62.5)	High ***n***= 27 (37.5)		
**Age**				
<60	14 (66.7)	7 (33.3)	0.220	0.790
≥60	31 (60.8)	20 (392)		
**Gender**				
Male	24 (54.5)	20 (45.5)	3.055	0.081
Female	21 (75)	7 (25)		
**Tumor grade**				
Low	18 (72)	7 (28)	1.475	0.308
High	27 (57.4)	20 (42.6)		
**T-Primary tumor**				
pT_a_	17 (85)	3 (15)		
pT_1_	19 (76)	6 (24)	16.345	0.001^*^
pT_2a_	5 (38.5)	8 (61.5)		
pT_2b_	4 (28.6)	10 (71.4)		
**Associated T**_**is**_				
Absent	26 (60.5)	17 (39.5)	0.189	0.805
Present	19 (65.5)	10 (34.5)		
**LVI**				
Absent	29 (74.4)	10 (25.6)	5.106	0.030^*^
Present	16 (48.5)	17 (51.5)		
**N-Regional lymph nodes**				
N_0_	9 (75)	3 (25)		
N_1_	3 (30)	7 (70)	6.372	0.041^*^
N_2_	1 (20)	4 (80)		
**M - Distant metastasis**				
M_0_	19 (82.6)	4 (17.4)	11.152	0.004^*^
M_1_	0 (0)	4 (100)		

*LVI* lymphovascular invasion, *x*^2^ chi-square test, *p <0.05

### CD34 as an illustrative marker for microblood vessels

CD34 brown cytoplasmic immunostaining was detected in the endothelial cells lining the microblood vessels. Intratumor microblood vessels were identified in all UC cases with the MVD value of all studied tumors being 30.14±4.79.

### Intratumor MVD of UC in relation to clinicopathological characteristics

**Fig. 3:**
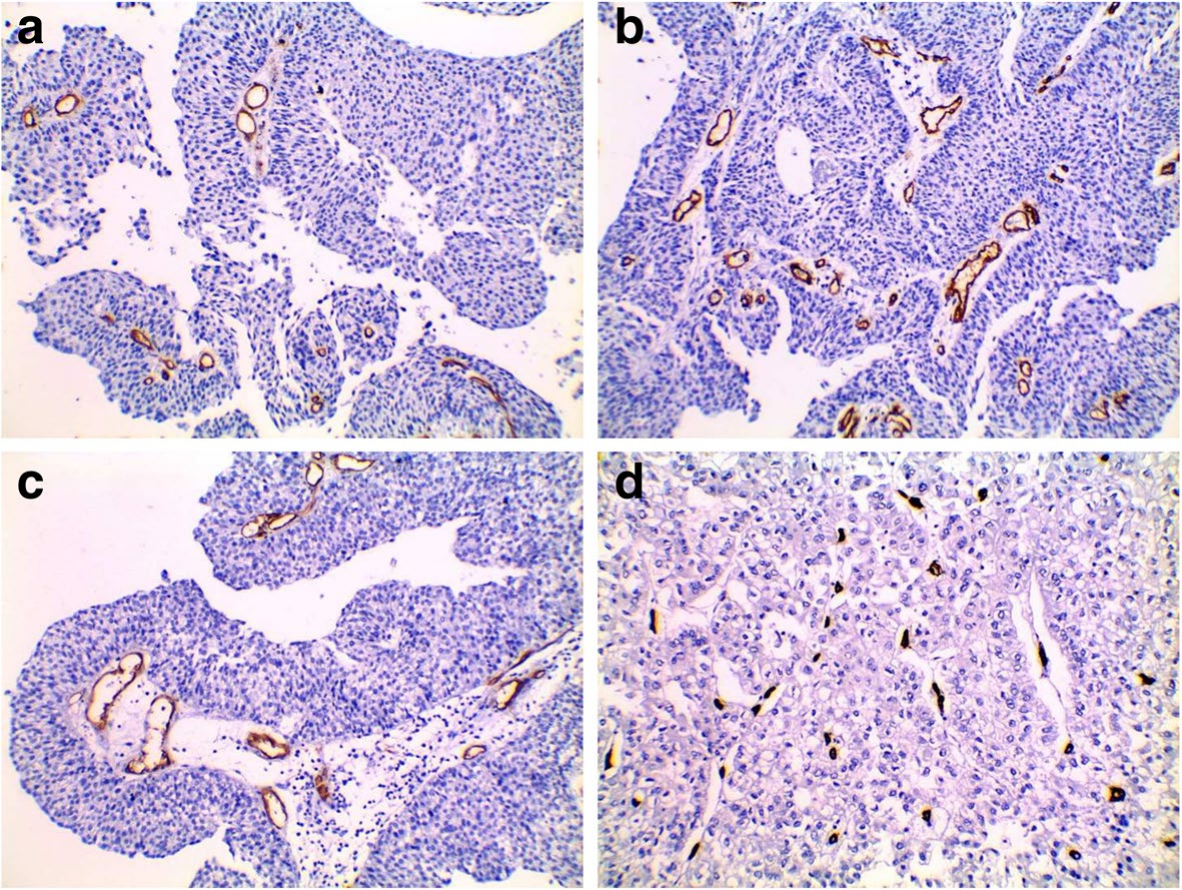
CD34 labeled intratumor micro blood vessels (×200) in: **a** low-grade pT_a_ UC, **b** low-grade pT_1_ UC, **c** high-grade pT_a_ UC, and **d** high-grade pT_2_ UC

Intratumor MVD was significantly higher in muscle-invasive urothelial carcinomas (pT_2a_ and pT_2b_) than in non-muscle-invasion tumors (pT_a_ and pT_1_) (33.72±3.45 and 27.96±4.18, respectively) (*p*<0.0001). Similarly, high intratumor MVD was significantly observed in tumors with LVI (31.94±4.89) compared to tumors without LVI (28.59±4.21) (*p*=0.003). Also, MVD increased significantly in UC associated with regional lymph node and/or distant metastasis with MVD being 35.70±1.34 and 37.25±0.17, respectively (*p*<0.0001 and 0.016, respectively). There was no significant difference in intratumor MVD between the two patient’s age groups, genders, tumor grades, or the presence of an associated carcinoma in situ component (*p*=0.413, 0.293, 0.134, and 0.176, respectively) (Table 4, Fig. 3).

**Table 4:** The relation between intratumor MVD and clinicopathological characteristics

	MVD±SD	***t test***	***p*** value
**Age**			
<60	29.39±4.89	-0.823	0.413
≥60	30.42±4.79		
**Gender**			
Male	30.60±5.06	-1.060	0.293
Female	29.37±1.69		
**Tumor grade**			
Low	28.96±5.17	-1.516	0.134
High	30.74±4.54		
**T-Primary tumor**			
pT_a_+ pT_1_	27.96±4.18	-6.023	<0.0001^*^
pT_2a_+ pT_2b_	33.72±3.45		
**Associated T**_**is**_			
Absent	29.49±4.26	-1.368	0.176
Present	31.06±5.47		
**LVI**			
Absent	28.59±4.21	-3.123	0.003^*^
Present	31.94±4.89		
**N-Regional lymph nodes**			
N_0_	26.53±3.28	-9.890	<0.0001^*^
N_1_+ N_2_	35.70±1.34		
**M - Distant metastasis**			
M_0_	30.65±5.02	-2.587	0.016^*^
M_1_	37.25±0.17		

*LVI* lymphovascular invasion, *MVD* microvessel density, *SD* standard deviation, *p <0.05

### Correlation between expression of GLUT4 and FAP

High FAP expression in CAFs was found to be linked to GLUT4 overexpression in neoplastic epithelial cells of UC with a significant positive correlation between these two biomarkers (*r*=0.426 and *p*<0.0001). Vice versa, low GLUT4 expression was significantly associated with low FAP immunoreactivity in CAFs (Table 5).

**Table 5:** Correlation between GLUT4 and FAP expression with intratumor MVD

	GLUT4 expression		FAP expression		r	***p*** value
	Low ***n***=40	High ***n***=32	Low ***n***=45	High ***n***=27		
**FAP expression**						
Low	33 (73.3)	12 (26.7)	**-**	**-**	0.462	<0.0001^*^
High	7 (25.9)	20 (74.1)				
**Intratumor MVD**	27.71±3.79	33.15±4.31	28.47±4.40	32.88±4.48		
***p*** **value**	<0.0001^*^		<0.0001^*^			

*MVD* microvessel density, *r* Spearman’s correlation, *p< 0.05

### Correlation between GLUT4 expression and intratumor MVD

A significant positive correlation was found between high GLUT4 expression in neoplastic epithelial cells and high intratumor MVD compared to UC with low GLUT4 expression (33.15±4.31 vs 27.71±3.79, *p*<0.0001) (Table 5).

### Correlation between FAP expression and intratumor MVD

High FAP immunostaining in CAFs was significantly correlated with higher intratumor MVD than those with low FAP expression (32.88±4.48 vs 28.47±4.40, *p*<0.0001) (Table 5).

## Discussion

Urothelial carcinoma of the urinary bladder is categorized into superficial and muscle-invasive primary tumors according to the depth of tumor invasion [22]. Even though UC responds better to chemotherapy than other subtypes of BC still, there is a subset of patients who develop chemoresistance after initial therapy improvement [23]. Therefore, it is very important to understand the biological behavior of the tumor for better selection of appropriate treatment on one hand and to predict the prognosis on the other hand. Although tumor stage and grade are considered important prognostic factors of UC [24], tumors with similar stages and grades may behave in different ways with various outcomes [25]. Hence, other prognostic factors may be necessary for predicting the prognosis of UC and improving its management.

The expression of GLUT4 and FAP in UC as prognostic markers and their relation to angiogenesis and other clinicopathological factors have not been addressed before. So, this work aims to study the expression of GLUT4 in neoplastic epithelial cells of UC in relation to FAP expression in CAFs and clinicopathological factors as well as tumor angiogenesis.

Reprogramming of energy metabolism and production is a hallmark of cancer. Cancer cells switch from oxidative phosphorylation to glycolysis for energy production to survive in a harsh microenvironment and to keep their high proliferative rate. This is called the Warburg effect, which imparts several genetic alterations in tumor cells, including overexpression of GLUTs and other molecules [26].

GLUT4 is an insulin-dependent glucose transporter. It has been considered as one of the important GLUTs for its critical role that might play in cancer cell survival and proliferation in several types of malignant tumors [10].

In the current study, it was found that high GLUT4 expression was significantly associated with high tumor grade, increased depth of tumor invasion, LVI, and regional lymph node metastasis. Similar to our findings but with different tissues, the high-glucose level in endometrial carcinoma cells through GLUT4 overexpression was found to increase tumor cell viability and invasion and decreased GLUT4 expression led to decrease tumor invasion [27]. Also, Chang et al. revealed that GLUT4 expression was higher in head and neck squamous cell carcinoma than in normal cells, and this higher expression was significantly related to poor prognosis and high recurrence rate [28]. In addition, GLUT4 overexpression in ER−, PR−/HER2+ breast carcinoma subtype was found to be significantly associated with high tumor grade, advanced TNM staging, and high proliferative Ki-67 index [29]. The adverse finding was reported by Zeng et al., who found that GLUT4 overexpression was associated with longer relapse-free survival in breast carcinoma patients than those with high GLUT4 expression [30].

The metabolism of cancer cells is different from that of normal cells, in contrast to normal cells which convert glucose into pyruvate for energy production via oxidative phosphorylation, cancer cells prefer to convert glucose to lactate even in the presence of oxygen, a process known as aerobic glycolysis [31]. Aerobic glycolysis not only generates ATP but also mediates the synthesis of metabolic molecules like nucleotides, lipids, and amino acids which are essential for rapid cell proliferation [32]. This explains why the glycolytic pathway is crucial for malignant cells. To maintain high glycolytic activity, the malignant cells increase glucose uptake through GLUTs overexpression [33]. Increased glucose influx is not only important for malignant cell growth and proliferation but also for epithelial to mesenchymal transition (EMT) which is an essential step before invasion and metastasis. It was found that GLUTs promote tumor invasion through activating several molecular pathways involved in EMT [34]. One of these important pathways is the PI3K-AKT-mTOR pathway which was found to be activated via GLUT4 [35]. All these findings support our results of GLUT4 overexpression association with adverse clinicopathological factors in the present study.

Malignant cell survival, proliferation, and invasion are not dependent on cancer cells alone; however, the pivotal stromal non-cancer cells play an important role in maintaining the demands of malignant cells essential for growth and invasion [36].

One of these cells is cancer-associated fibroblasts (CAFs), the most widely distributed abundant stromal cells of several solid tumors, and one of the most important stromal cells mediating tumor-stroma link supporting cancer cells’ survival and proliferation [37].

In this study, the expression of FAP in CAFs was investigated in relation to clinicopathological factors of UC, and it was found that high FAP immunostaining was significantly associated with increased depth of tumor invasion, LVI, regional lymph node invasion, and distant metastasis. In concordance with our findings, the relation between FAP overexpression in CAFs, on the one hand, and poor overall survival and lymph node metastasis in solid tumors on the other hand has been confirmed in a meta-analysis study [38]. Also, Calvete et al. showed that FAP expression in activated fibroblasts was significantly associated with high pT stage and aggressive UC [39]. An additional study on muscle-invasive and non-muscle-invasive UC proved that FAP expression in CAFs was associated with poor overall survival [15]. Similarly, Muilwijk et al. found that FAP expression in high-grade pT_1_ UC was considered a poor prognostic factor [40].

Regarding the correlation between GLUT4 expression in neoplastic epithelial cells and FAP expression in CAFs, a significant positive correlation was found as 74.1% of tumors with GLUT4 overexpression exhibited high FAP expression in CAFs too.

CAFs play several crucial roles in tumorigenesis, cancer growth, proliferation, survival, and metastasis [41]. Olumi et al. found that when non-tumorigenic prostate epithelial cells were transplanted into nude mice without CAFs, they proliferated without tumorigenic capacity; but when they were co-transplanted with CAFs, a tumor was gradually formed supporting the role of CAFs in tumorigenesis [42]. CAFs further stimulate the proliferation of malignant cells by secreting several growth stimulating factors and cytokines [43]. CAFs were found to be capable of switching on alternative metabolic pathways to buffer the products of anaerobic glycolysis, modifying the tumor microenvironment, and facilitating the survival of cancer cells in unfavorable environments such as low pH and hypoxia [44]. This explains the finding of a positive correlation between the GLUT4 and FAP expression in the present study. Besides, CAFs can facilitate cancer invasion and metastasis through secreting substances such as proteinases which degrade the ECM. Also, CAFs promote EMT of malignant epithelial cells through secreting several mediators or by direct cell-to-cell contact [45].

Angiogenesis is a multistep process, which involves endothelial cell proliferation, migration, and differentiation into capillaries. Studies of tumor biology reveal a complex network between tumor cells, stromal cells, and endothelial cells are involved in process of angiogenesis [46]. MVD is used to assess tumor vasculature using an antibody that identifies endothelial cells. Increased MVD has been correlated with adverse pathological factors and poor outcomes in patients with bladder cancer [21, 47].

In the present study, the relation between the intratumor MVD, using CD34 as an illustrative marker for micro blood vessels, and clinicopathological factors was studied. It was found that high intratumor MVD was significantly detected in UC with advanced pT stage, LVI, regional lymph node invasion, and distant metastasis. Alike, a meta-analysis study showed that high intratumor MVD has been correlated positively with adverse pathological findings and poor outcomes in patients with bladder cancer [48]. Only a minority of studies did not address that association [49, 50].

The correlation between GLUT4 and FAP expression with intratumor MVD in UC was investigated in the current work. A significant positive correlation was detected between GLUT4 overexpression in malignant epithelial cells, high FAP expression in CAFs, and increased intratumor MVD.

As mentioned previously, tumor cells exhibit a high tendency for aerobic glycolysis even in the presence of oxygen [5]. They internalize large amounts of glucose by overexpressing GLUTs converting them to pyruvate for ATP production. Pyruvate is then converted to lactate which is rapidly exported outside the malignant cells to maintain intracellular pH homeostasis and prevent apoptosis. Lactate is then taken up by adjacent endothelial cells and CAFs in the tumor microenvironment. High lactate dependence of the CAFs and endothelial cells stimulates the production of several cytokines, growth, and angiogenic factors which further promotes tumor growth, angiogenesis, and invasion [51]. Moreover, it was found that reduced glycolysis in in vitro UM-UC-3 bladder cancer cell lines by decreasing glucose uptake has led to decrease lactate production and reduced cell viability in these cells [52]. Furthermore, lactate dehydrogenase (LDH) overexpression, which converts lactate into NADH and NAD for continued glycolytic activity, has been identified as an independent prognostic factor for bladder cancer and is associated with enhancement of EMT and poor outcomes in patients with bladder cancer [53]. All these facts interpret the positive correlation that has been found between high GLUT4 and FAP expression and increased intratumor MVD in UC.

Finally, the complex metabolic pathways particularly the glycolytic pathway and its resultant metabolites required for cancer cells as well as the non-cancer associated stromal cells notably CAFs require multiple therapeutic agents targeting different aspects of the tumorigenic process. This new strategic treatment plan could improve the outcome of patients with UC.

## Conclusions

From our results, it was concluded that GLUT4 expression in malignant epithelial cells of UC, as well as FAP expression in CAFs, were significantly associated with adverse clinicopathological factors namely high tumor grade, advanced pT stage, LVI, regional lymph node invasion, and distant metastasis in addition to increased intratumor MVD. Therefore, the role of reprogrammed cellular metabolism, aerobic glycolysis, and increased glucose uptake, as well as the synergistic action between tumor cells and CAFs in the proliferation, progression, and prognosis of UC, should be considered in designing anticancer drugs directed to disrupt the glucose metabolism in tumor cells together with targeting the tumor-stromal interactions to improve the outcome of patients with UC.

## Data Availability

The original contributions presented in the study are included in the article, and further inquiries can be directed to the corresponding author.

## Abbreviations

ASMA: Alpha-smooth muscle actin
ATP: Adenosine triphosphate
BC: Bladder carcinoma
CAFs: Cancer-associated fibroblasts
ECM: Extracellular matrix
EMT: Epithelial-mesenchymal transition
FAP: Fibroblast activation protein
FSP: Fibroblast specific protein
GLUT: Glucose Transporter
GLUT4: Glucose Transporter 4
H&E: Hematoxylin &Eosin
LDH: Lactate dehydrogenase
LVI: Lymphovascular invasion
MVD: Microvessel density
NAD: Nicotinamide adenine dinucleotide
NADH: Nicotinamide adenine dinucleotide hydrogen
PDGFR α, -ß: Platelet-derived growth factor receptor-alpha and -beta
SGLT: Sodium-coupled glucose co-transporter
TURBT: Transurethral resection of bladder tumor
UC: Urothelial carcinoma
